# MSA-Net: A Precise and Robust Model for Predicting the Carbon Content on an As-Received Basis of Coal

**DOI:** 10.3390/s24144607

**Published:** 2024-07-16

**Authors:** Yinchu Wang, Zilong Liu, Feng Chen, Xingchuang Xiong

**Affiliations:** 1 National Institute of Metrology, Beijing 100029, China; wangych@nim.ac.cn (Y.W.); liuzl@nim.ac.cn (Z.L.); xaf_love_life@163.com (F.C.); 2 Key Laboratory of Metrology Digitalization and Digital Metrology for State Market Regulation, Beijing 100029, China

**Keywords:** coal, carbon content as received, carbon content prediction, MLP, attention mechanism

## Abstract

The carbon content as received (*C_ar_*) of coal is essential for the emission factor method in IPCC methodology. The traditional carbon measurement mechanism relies on detection equipment, resulting in significant detection costs. To reduce detection costs and provide precise predictions of *C_ar_*s even in the absence of measurements, this paper proposes a neural network combining MLP with an attention mechanism (MSA-Net). In this model, the Attention Module is proposed to extract important and potential features. The Skip-Connections are utilized for feature reuse. The Huber loss is used to reduce the error between predicted *C_ar_* values and actual values. The experimental results show that when the input includes eight measured parameters, the MAPE of MSA-Net is only 0.83%, which is better than the state-of-the-art Gaussian Process Regression (GPR) method. MSA-Net exhibits better predictive performance compared to MLP, RNN, LSTM, and Transformer. Moreover, this article provides two measurement solutions for thermal power enterprises to reduce detection costs.

## 1. Introduction

Excessive global CO_2_ emissions will exacerbate the greenhouse effect, leading to extreme weather events around the world and greatly impacting human survival and development [1]. Although many researchers have conducted research on carbon emissions [2], carbon reduction [3], and other related issues [4,5], the global impact of the greenhouse effect is still intensifying. Energy-related CO_2_ emissions are a significant contributor to global CO_2_ emissions. According to the 2022 CO_2_ emissions statistics published by the International Energy Agency (IEA) in March 2023, global energy-related CO_2_ emissions were higher than 36.8 Gt in 2022, an increase of approximately 0.9% compared to 2021 [6]. As a major carbon emitter, China had energy-related CO_2_ emissions of 10.2 Gt in 2022 [6], accounting for approximately 27.7% of global energy-related CO_2_ emissions. Among them, the CO_2_ emissions generated by coal consumption in the thermal power industry account for nearly 40%. Therefore, China has focused on CO_2_ emission management in the thermal power industry, which is a highly energy-consuming industry, and has conducted extensive research on this topic [7,8].

In the carbon accounting of the thermal power industry, in order to ensure the accuracy of CO_2_ emission data, multiple parameters need to be measured. According to GB/T 32151.1-2015 “Greenhouse Gas Emission Accounting and Reporting Requirements Part 1: Power Generation Enterprises” [9], and according to the “Guidelines for Enterprise Greenhouse Gas Emission Accounting and Reporting” issued by the Ministry of Ecology and Environment in 2022 [10], the measured parameters related to CO_2_ emissions generated by coal combustion mainly include furnace coal weight (*FC*), total moisture (*M_t_*), carbon content as received (*C_ar_*), net calorific value as received (*NCV*), moisture (*M_ad_*), total sulfur (*S_t_*_,*ad*_), hydrogen (*H_ad_*), ash (*A_ad_*), volatile matter (*V_ad_*), and fixed carbon (*FC_ad_*). The last six parameters are expressed as percentages on an air-dried basis. *FC*, *C_ar_*, and the carbon oxidation rate *OF* are used for CO_2_ emission calculation. Therefore, the measurement of *C_ar_*s directly affects the carbon accounting results. If the *C_ar_*s are not measured or the measurements do not meet the requirements, it is necessary to use the default value of carbon content per unit calorific value (*CC*) and the *NCV* for conversion. Although the default value of *CC* had been lowered from 0.03356 tC/t to 0.03085 tC/t [11], the calculated CO_2_ emissions based on this default value are still relatively high compared to the actual carbon emissions, resulting in thermal power enterprises bearing additional compliance costs.

In order to ensure the accuracy of carbon accounting, reduce detection costs, and allocate reasonable compliance costs in thermal power enterprises, many researchers have considered how to make reasonable predictions of carbon content when the measured data are incomplete during the statistical period. With the improvement in carbon accounting methods of thermal power enterprises and the further clarification of measured parameters, carbon content has become a key parameter for carbon accounting based on existing measured parameters. To achieve prediction of carbon content, Zhang et al. combined an attention mechanism with bidirectional ResNet-LSTM to propose the ABRF model [12]. However, this model is only applicable to a single type of coal, which is inconsistent with the actual situation where thermal power plants usually use multiple coal blends for combustion [13,14], resulting in poor applicability of this model. Deng et al. proposed a simulated annealing differential evolution (SADE) neural network to predict the composition of coal [15]. However, this method can only predict the fixed carbon content and cannot further predict the carbon content. Guan et al. proposed a method for predicting the carbon content of coal powder using Double Spectral Correction-Partial-Least-Squares (DCS-PLS) [16]. However, the prediction relies on the collected signals of Laser-Induced Breakdown Spectroscopy (LIBS). Meanwhile, signal acquisition is still required in applications, which increases the detection costs. Jo et al. proposed an ANN-based method for predicting coal elements, but the model design of this method is too simple and the prediction accuracy is low [17]. In addition, Ceylan et al. utilized moisture, ash, volatile matter, and fixed carbon as model inputs and the Gaussian Process Regression (GPR) model to predict elemental carbon content [18]. However, the selection of kernel functions and hyper-parameters is difficult and requires extensive experience-based optimization. Yin et al. used Multi-Layer Perceptron (MLP) for carbon content prediction and applied it to CO_2_ emission calculation [19]. Although good prediction accuracy was achieved, the consideration of input parameters in model design was still incomplete, such as *M_t_*, *M_ad_*, *S_t_*_,*ad*_, and *H_ad_*, which were also correlated with carbon content. This problem limits the prediction accuracy of the models. Moreover, if more parameters are utilized, the models need to fully explore the interaction mechanism between each parameter and have the ability to pay attention to important and potential features (formed by Linear layer mapping).

For model input with all parameters, firstly, a qualitative analysis was conducted on the coal parameters related to the *C_ar_*, and the input parameters for *C_ar_* predicting were determined. Secondly, after using all parameters, this paper introduces the attention mechanism [20] into the model design and proposes an Attention module for predicting *C_ar_* while paying attention to important and potential features. Then, feature reuse was achieved through Skip-Connections [21] to improve feature utilization efficiency and form the MSA-Net model. Next, in order to improve the convergence of model training, we chose Huber loss as the loss function. Finally, to validate the effectiveness of the proposed method, we preprocessed the collected coal parameters and constructed the training and predicting datasets. Through quantitative and qualitative comparative experiments, the effectiveness and reliability of MSA-Net were verified. Meanwhile, through comparative results under different data split methods, the corresponding solutions were proposed for the practical application of MSA-Net in thermal power enterprises.

## 2. Analysis of Coal Carbon Content as Received

The main parameters for carbon accounting in thermal power enterprises are the consumption of coal and the carbon content of the base element received from coal. To predict the *C_ar_*, which is a key carbon accounting parameter, this paper further analyzes the *C_ar_* based on the relationship between the dry carbon content *C_d_* and various parameters in proximate analysis and ultimate analysis methods [22]. According to the calculation method

(1) $$C_{d}=35.411-0.341A_{d}-0.199V_{d}-0.412S_{t,d}+1.632Q_{gr,d},$$

where *C_d_* is dry basis carbon content, *A_d_* is dry basis ash, *V_d_* is dry basis volatile matter, *S_t_*_,*d*_ is dry basis total sulfur and high calorific value *Q_gr_*_,*d*_, *α*_0_ = 35.411, *α*_1_ = −0.341, *α*_2_ = −0.199, *α*_3_ = −0.412, *α*_4_ = 1.632. The *C_d_* is correlated with *A_d_*, *V_d_*, *S_t_*_,*d*_, and *Q_gr_*_,*d*_. Firstly, considering that the measurements are mostly based on air-dried basis, it is necessary to achieve benchmark conversion between air-dried basis and dry basis based on the Mad for prediction. Secondly, due to the 100% mass fraction of *M_ad_*, *A_ad_*, *V_ad_*, and *FC_ad_*, when *M_ad_*, *A_ad_*, and *V_ad_* are measured, *FC_ad_* can be calculated. Therefore, *FC_ad_* also has a certain correlation with *C_d_*. Then, due to the influence of early CO_2_ emission calculation methods, the calorific value of coal measured by Chinese thermal power enterprises is the *NCV*. Significantly, *Q_gr_*_,*d*_ can combine with *M_ad_* and *H_ad_* to convert into *NCV*. Therefore, *NCV* and *H_ad_* should also be taken into consideration. Finally, the basis conversion between dry basis and as received requires *M_t_*, which should also be taken into account.

In summary, considering the principle of coal detection and analysis in China [23,24,25], this paper takes *M_t_*, *M_ad_*, *A_ad_*, *V_ad_*, *FC_ad_*, *H_ad_*, *S_t_*_,*ad*_, and *NCV* as inputs to the prediction model. Furthermore, *C_ar_* is set as output to explore the construction method of the prediction model. We used a total-moisture analyzer 5E-MW6510 (Automatic Moisture Analyser produced from Changsha Kaiyuan Instruments Co., Ltd., Changsha, China) to determine *M_t_* according to GB/T 211-2017 [26]. *M_ad_*, *A_ad_*, *V_ad_,* and *FC_ad_* are measured by a proximate analysis instrument 5E-MAG6700 (Proximate Analyzer produced from Changsha Kaiyuan Instruments Co., Ltd., Changsha, China) according to GB/T 212-2008 [27]. *S_t_*_,*ad*_ is measured by an automatic coulomb sulfur analyzer 5E-AS3200B (Automatic Coulomb Sulfur Analyzer produced from Changsha Kaiyuan Instruments Co., Ltd., Changsha, China) according to GB/T 214-2007 [28]. *C_ad_* and *H_ad_* are measured by a carbon–hydrogen–nitrogen elemental analyzer 5E-CHN2200 (C/H/N Elemental Analyzer produced from Changsha Kaiyuan Instruments Co., Ltd., Changsha, China)according to GB/T 476-2008 [29]. *NCV* and *Q_gr_*_,_*_ad_* are measured by a calorimeter 5E-C5500 (Automatic Calorimeter produced from Changsha Kaiyuan Instruments Co., Ltd., Changsha, China) according to GB/T 213-2008 [25].

## 3. Coal Carbon Content as Received Prediction Model

In this section, the eight parameters introduced in Section 2 are first used as model inputs to provide comprehensive reference parameters for *C_ar_* prediction. Secondly, in order to extract important and potential features, an Attention module suitable for predicting *C_ar_* in coal combustion is proposed. By combining and mapping the input or intermediate layer results, it outputs features suitable for predicting *C_ar_*. Then, the proposed Attention module is added to the model design, and Skip-Connections are used for feature reuse to build the MSA-Net model. Subsequently, the Huber loss is adopted as the loss function to train the MSA-Net model until convergence. Finally, the prediction of *C_ar_* in coal combustion is achieved.

### 3.1. Attention Module

**Figure 1:**
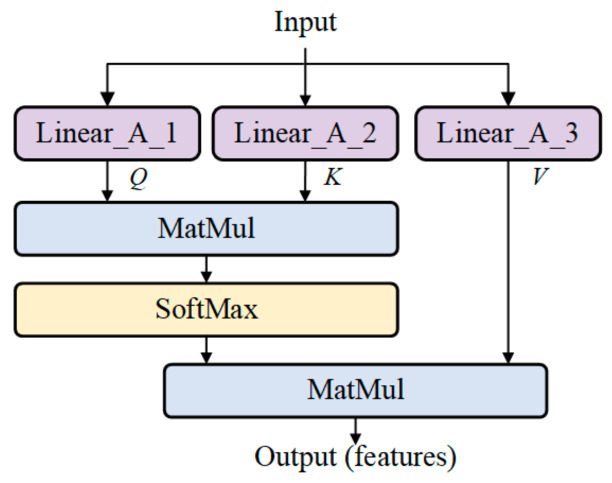
The structure of Attention Module.

In this paper, we refer to the design of attention mechanism in Natural Language Processing (NLP) [30] and construct an Attention module for predicting *C_ar_*. The structure is shown in Figure 1. For input parameters or mapped features, three Linear layers (Linear_A_1, Linear_A_2, Linear_A_3) are used to obtain corresponding *Q* (query), *K* (key), and *V* (value). The similarities between *Q*s and *K^T^*s are calculated using matrix multiplication. We obtain these similarities and normalize them using SoftMax(·) to obtain the weights. By weighted summing the weights and *V*, we obtain the output feature *F*, which is calculated as

(2) $$F=SoftMax\left(QK^{T}\right)V,$$

### 3.2. MSA-Net Model

**Figure 2:**
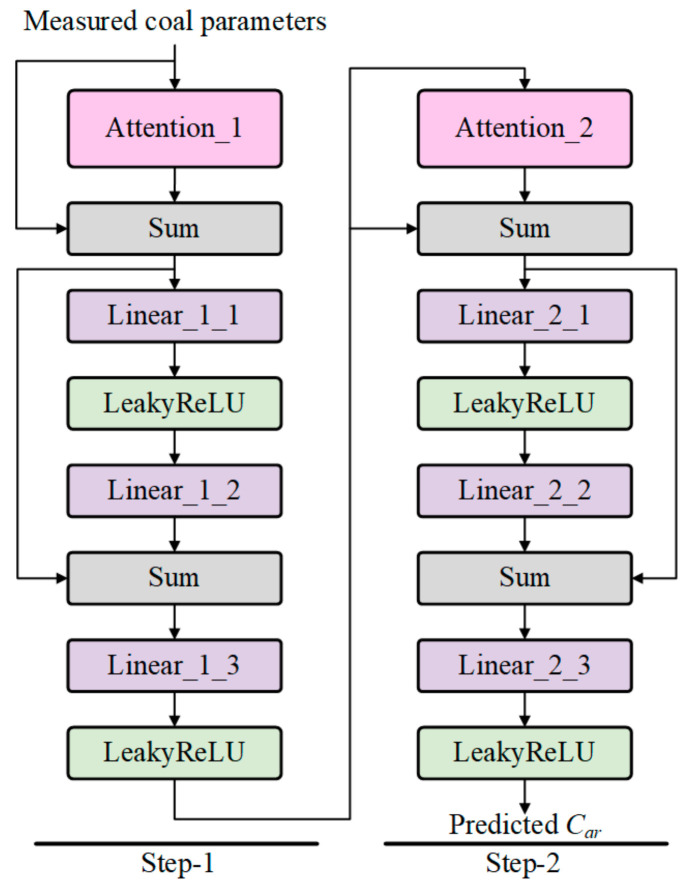
MSA-Net structure.

This paper introduces the proposed Attention module into the model based on the basic structure of MLP. At the same time, to better extract important and potential features, Skip-Connections are used to sum shallow features with mapped features, enabling feature reuse and guiding the synthesis of new features. The network structure is shown in Figure 2.

MSA-Net consists of two similar structures (Step-1 and Step-2). In Step-1, the input is the measured parameters of coals. The potential features are generated from the Attention module. We utilize 3 linear layers and 2 activation layers for feature integration and mapping, Skip-Connections are added and feature reuse is achieved through Sum operation. The output is the mapped feature. In Step-2, the input is the mapping feature obtained from Step 1, and its structure is similar to that of Step 1. But the output dimension of the last Linear layer is adjusted to 1 for *C_ar_* prediction. If *N*-measured parameters are used as input to the model, the corresponding trainable parameters are shown in Table 1. In the experiments, *M* is set to 128 as the mapping dimension of the hidden layer. Meanwhile, *α* is set to 0.01 for LeakyReLU.

### 3.3. Loss Function

To ensure the robustness of the model to outliers during training and reduce its sensitivity to outliers, while also ensuring that the gradient gradually decreases as the loss value approaches its minimum, this paper uses the Huber loss *L_δ_*(·) as the loss function for model training. The formula is

(3) $$L_{\delta }\left(y,y^{\prime }\right)=\left\{\begin{array}{cc} \frac{1}{2}{\left(y-y^{\prime }\right)}^{2} & \left|y-f\left(x\right)\right|\leq \delta \\ \delta \left|y-y^{\prime }\right|-\frac{1}{2}\delta^{2} & otherwise \end{array}\right.,$$

where *y* represents the measured *C_ar_*, and *y*′ represents the algorithms or model prediction results. In the experiments, *δ* is set to 1.0.

## 4. Training and Predicting Datasets and Evaluation Indicators

### 4.1. Complete Dataset Production

**Figure 3:**
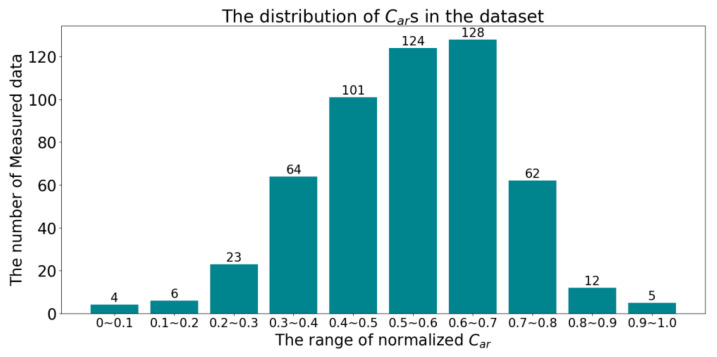
The distribution of normalized *C_ar_*s.

We collected the measured data of a typical thermal power enterprise from 1 September 2022 to 31 August 2023. This enterprise has two generating units. The daily coal quality analysis and parameter measurements are performed on the coal consumed by both units. Due to shutdowns and routine maintenance, a total of 687 data points were collected. Each data point contains nine parameters: *M_t_*, *M_ad_*, *A_ad_*, *V_ad_*, *FC_ad_*, *H_ad_*, *S_t_*_,*ad*_, *NCV,* and *C_ar_*. After removing missing and abnormal values, a total of 529 pieces of data were sorted out. We performed maximum and minimum normalization on all measured data in the dataset, and the distribution of normalized *C_ar_* is shown in Figure 3.

### 4.2. Training and Predicting Datasets Split Methods

In the experiments, training and prediction were performed according to three rules of the dataset created in Section 4.1, according to three rules in the experiment: data partitioning based on stratified sampling, data partitioning based on odd or even months, and data partitioning based on odd or even dates.

#### 4.2.1. Data Partitioning Based on Stratified Sampling

**Figure 4:**
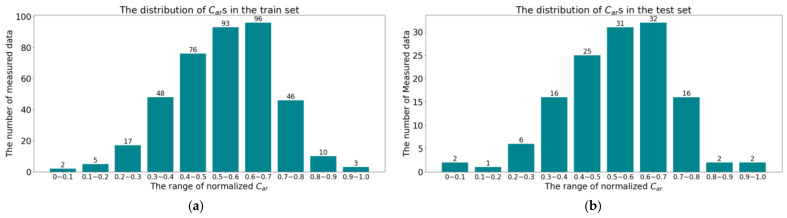
The distribution of *C_ar_*s after stratified sampling: (**a**) The distribution of *C_ar_*s on the training set; (**b**) The distribution of *C_ar_*s on the testing set.

In the experiments of Section 5.2 and Section 5.3, 75% of the data in the dataset was divided into a training set and 25% into a testing set using stratified sampling. The distribution of *C_ar_*s for this partitioning method is shown in Figure 4.

#### 4.2.2. Data Partitioning Based on Odd or Even Months

**Figure 5:**
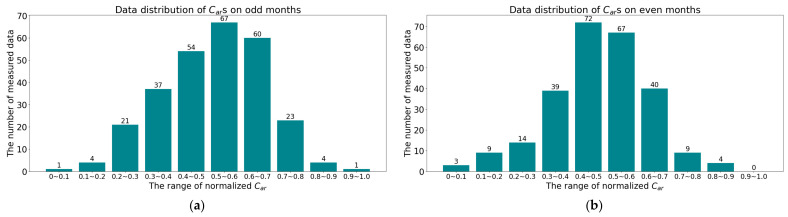
The distribution of *C_ar_*s by months: (**a**) The distribution of *C_ar_*s on odd months; (**b**) The distribution of *C_ar_*s on even months.

In the experiments of Section 5.4, we divided each data point into odd and even numbers based on the actual measurement date of each dataset, with a total of 272 odd-month data and 257 even-month data. The distribution of *C_ar_* for odd and even months is shown in Figure 5.

#### 4.2.3. Data Partitioning Based on Odd or Even Days

**Figure 6:**
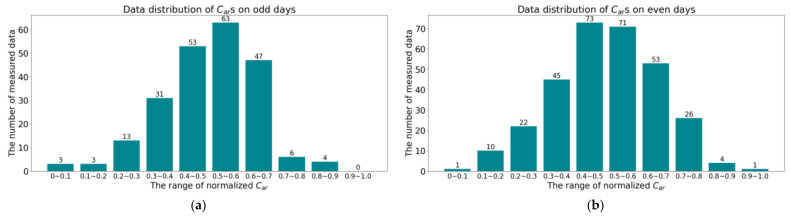
The distribution of *C_ar_*s by months: (**a**) The distribution of *C_ar_*s on odd days; (**b**) The distribution of *C_ar_*s on even days.

In the experiments of Section 5.5, we further divided the collected 529 data points into odd and even dates, with a total of 223 odd-day data and 306 even-day data. The distribution of *C_ar_* data for odd days and even numbered days is shown in Figure 6.

### 4.3. Evaluation Metrics

This paper quantitatively compared the proposed method with existing methods using seven evaluation indicators in the experiment. These evaluation metrics are as follows.

1. Mean Absolute Error (MAE)

(4) $$MAE\left(y,y^{\prime }\right)=\frac{1}{n}\sum_{i=1}^{n}\left|y-y^{\prime }\right|,$$

2. Root Mean Square Error (RMSE)

(5) $$RMSE\left(y,y^{\prime }\right)=\sqrt{\frac{1}{n}\sum_{i=1}^{n}{\left(y_{i}-y_{i}^{\prime }\right)}^{2}},$$

3. Mean Absolute Percentage Error (MAPE)

(6) $$MAPE\left(y,y^{\prime }\right)=\frac{1}{n}\sum_{i=1}^{n}\frac{\left|y_{i}-y_{i}^{\prime }\right|}{y_{i}}\times 100\%,$$

4. Coefficient of Determination (R^2^)

(7) $$R^{2}\left(y,y^{\prime }\right)=1-\frac{\sum_{i=1}^{n}{\left(y_{i}-y_{i}^{\prime }\right)}^{2}}{\sum_{i=1}^{n}{\left(y_{i}-\bar{y}\right)}^{2}},$$

5. Pearson Correlation Coefficient (PCC)

(8) $$PCC\left(y,y^{\prime }\right)=\frac{cov\left(y,y^{\prime }\right)}{\sigma_{y}\sigma_{y^{\prime }}},$$

6. Concordance Correlation Coefficient (CCC)

(9) $$CCC\left(y,y^{\prime }\right)=\frac{2PCC\left(y,y^{\prime }\right)\sigma_{y}\sigma_{y^{\prime }}}{\sigma_{y}^{2}+\sigma_{y^{\prime }}^{2}+{\left(\mu_{y}-\mu_{y^{\prime }}\right)}^{2}},$$

7. Explained Variance (Evar)
  (10) $$Evar\left(y,y^{\prime }\right)=1-\frac{\sigma_{y-y^{\prime }}^{2}}{\sigma_{y}^{2}},$$
  where *δ_x_* represents the standard deviation of *x*, and *μ_x_* represents the mean of *x*. The values of MAE, RMSE, and MAPE are all greater than 0, where smaller values indicate better prediction performance. The values of R^2^, PCC, CCC, and Evar are all between 0 and 1. The closer the value is to 1, the better the model’s prediction performance.

## 5. Experiments and Analysis

In this section, we compared the performance of MSA-Net with existing models. In addition, we also conducted ablation experiments to analyze the effects of different parts and combinations in MSA-Net. Finally, we conducted application research under different data partitions, providing an effective solution for the practical application of thermal power enterprises when the measured data are incomplete, and further testing the robustness of our trained model.

### 5.1. Implementation Details

The MSA-Net proposed in this paper is implemented on PyTorch-1.13.0. We conducted model training and testing on NVIDIA GeForce RTX 3070Ti (Graphics Processing Unit produced from NVIDIA Corporation, Santa Clara, CA, USA) with 8 GB of memory. In all experiments, we used the Adam optimizer [31] for model optimization, with *β*_1_ = 0.9, *β*_2_ = 0.999, and the batch size was set to 16. The model is trained for 240 epochs. The learning rate warm-up strategy [32] was employed during training. The first 40 epochs were the warm-up phase, followed by 200 epochs of learning rate decay. The learning rate *lr* in each epoch was calculated as

(11) $${lr}_{epoch}=\left\{\begin{array}{cc} \frac{epoch}{40}\times {lr}_{max} & epoch\leq 40\\ {{lr}_{epoch-1}}^{1.001} & epoch>40 \end{array}\right.,$$

where *lr_max_* is the maximum learning rate in the experiment, which is set to 0.005.

In comparison experiments, we considered two input modes. One refers to the four parameters of the industrial analysis method, including *M_ad_*, *A_ad_*, *V_ad_*, and *Q_gr_*_,*ad*_. Another type includes all eight parameters. Since the training results of neural network-based methods (such as RNN [33], LSTM [34], MLP [19], and MSA-Net) vary under different parameter initializations, for the same model, we used ten different parameter initialization methods to train the model to convergence using the above parameters. We then statistically evaluated the 10 different prediction results based on the evaluation indicators in Section 4.2. In experiments Section 5.2 and Section 5.4, to verify the effectiveness of the Attention Module, in the MLP, the Attention Module was replaced with a linear layer of size *N* × *N*.

All comparative experiments were conducted using two model input methods. Referring to the correlation between measured parameters and elemental carbon content in the industrial analysis method in Section 2, the model input of the first method consists of four parameters: *M_ad_*, *A_ad_*, *V_ad_*, and *Q_gr_*_,*ad*_. *Q_gr_*_,*ad*_ can be calculated based on *NCV*, *H_ad_*, *M_t_*, and *M_ad_*. The calculation method is

(12) $$Q_{gr,ad}=\left(NCV+23M_{t}\right)\times \frac{100-M_{ad}}{100-M_{t}}+206H_{ad}$$

The model input for the second method includes eight parameters: *M_t_*, *M_ad_*, *A_ad_*, *V_ad_*, *FC_ad_*, *H_ad_*, *S_t_*_,*ad*_, and *NCV*.

### 5.2. Models Performance Experiments and Analysis

To verify the effectiveness of the proposed model, we compared MSA-Net with existing methods. The comparison results are shown in Table 2. When the input is four parameters, MSA-Net achieved optimal results on all seven metrics. Compared to the GPR model (SOTA), the MSA-Net model achieved lower prediction errors. The MAE decreased by 2.67% (from 4.86 to 4.73) and the RMSE decreased by 2.81% (from 6.77 to 6.58). Compared to the MLP model, the MAE decreased by 6.34% (from 5.05 to 4.73) and the RMSE decreased by 4.78% (from 6.91 to 6.58).

When the inputs are eight parameters, MSA-Net also achieved the best performance on seven metrics. Compared to the GPR model (SOTA), the MAE decreased by 9.36% (from 5.02 to 4.55) and the RMSE decreased by 10.92% (from 6.96 to 6.20). Compared to the MLP model, the MAE decreased by 6.95% (from 4.89 to 4.55) and RMSE decreased by 6.91% (from 6.66 to 6.20). Due to the Attention module’s focus on important or potential features, when all parameters are used as inputs, MSA-Net can effectively capture the important and potential features, further improving the prediction accuracy. The MAE is reduced by 3.81% (from 4.73 to 4.55), and the RMSE is reduced by 5.78% (from 6.58 to 6.20).

**Figure 7:**
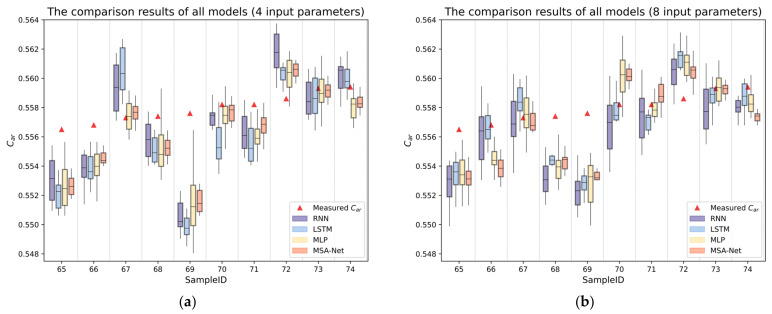
Visual comparison of partial prediction results in the test set: (**a**) The comparison results of different models (input four parameters); (**b**) The comparison results of different models (input eight input parameters).

Due to the differences in training results of neural network models under different parameter initializations, we also counted the prediction results under different parameter initializations. After sorting out the prediction results of some samples, a box plot is drawn as shown in Figure 7. When the inputs are four or eight parameters, the uncertainty of MSA-Net prediction results is significantly smaller than RNN, LSTM, and MLP models, and the median is also closer to the measured true value. We further summarized the mean and standard deviation of the prediction errors on the test set, as shown in Table 3. When given four input parameters, MSA-Net achieved the best performance on all seven evaluation metrics in terms of both the mean and standard deviation. When given eight input parameters, MSA-Net achieved the best performance on all seven evaluation metrics in terms of the mean. In addition, it achieved the lowest standard deviation on three metrics. Overall, MSA-Net not only achieves good prediction accuracy but also has stable prediction results under different parameter initializations after training.

### 5.3. Ablation Analysis

To verify the effectiveness of the proposed modules, we conducted ablation experiments as shown in Table 4. By adopting Huber loss on the MLP (Model A), the MAE can be reduced by 1.78% (from 5.05 to 4.96). By adding one Attention module (Model B), the MAE is reduced by 1.01% (from 4.96 to 4.91). With two Attention modules (Model C), the MAE is further reduced by 1.81% (from 4.96 to 4.87). However, when increasing the number of Attention modules to three (Model D), the prediction accuracy of the model has significantly decreased. By incorporating Skip-Connections on the basis of two Attention modules, the proposed MSA-Net can further reduce the MAE by 2.87% (from 4.87 to 4.73). When the number of input parameters is increased from four to eight, the MAE is reduced by 3.81% (from 4.73 to 4.55).

### 5.4. Model Testing Experiments on Dividing Datasets on Odd and Even Months

We quantitatively compared MSA-Net with RNN, LSTM, and MLP on seven evaluation metrics, and presented the mean and standard deviation of each metric under 10 different parameter initialization methods. We conducted this part of the experiment in two ways. The first method was to train the model using odd-month data and make predictions on even-month data. The quantitative comparison results are shown in Table 5. The second method is to use even-month data for model training and predict odd-month data. The quantitative comparison results are shown in Table 6.

By comparing the prediction errors of different models, in Table 5, when the inputs are four parameters, the MSA-Net model reduces RMSE by 12.97% (from 7.40 to 6.44) compared to the RNN model. Compared with the LSTM model, RMSE decreases by 15.15% (from 7.59 to 6.44), and RMSE decreases by 8.00% (from 7.00 to 6.44) compared to the MLP model. The MSA-Net model reduces RMSE by 3.88% (from 6.70 to 6.44) compared to the Transformer model. When the inputs are eight parameters, taking the RMSE index as an example, MSA-Net decreased by 14.55% (from 7.49 to 6.40) compared to RNN, 18.26% (from 7.83 to 6.40) compared to LSTM, 12.81% (from 7.34 to 6.40) compared to MLP, and 2.44% (from 6.56 to 6.40) compared to Transformer. When the number of input parameters increased from four to eight, the MAE of the MSA-Net model decreased by 1.87% (from 4.81 to 4.72). In Table 6, when inputting four parameters, the MSA-Net model reduces RMSE by 11.19% (from 6.97 to 6.19) compared to the RNN model, and reduces RMSE by 11.32% (from 6.98 to 6.19) compared to the MLP model. The MSA-Net model reduces RMSE by 7.06% (from 6.66 to 6.19) compared to the Transformer model. When the number of input parameters increased from four to eight, the accuracy of the MSA-Net model slightly decreased, and the MAE increased by 0.70% (from 4.31 to 4.34).

By comparing the predictive stability of different models, in Table 5 and Table 6, when the inputs are four or eight parameters, MSA-Net has the smallest standard deviation on seven indicators compared to RNN, LSTM, MLP, and Transformer, indicating that MSA-Net can predict more stably under different parameter initialization conditions.

In summary, for the thermal power enterprises, within a one-year statistical period, they can measure the elemental carbon in the first nine months according to the requirements and use these measured data for training the MSA-Net model. In the last three months, they only need to measure the *M_ad_*, *A_ad_*, *V_ad_*, and *Q_gr_*_,*ad*_ to predict the *C_ad_* using the trained MSA-Net model. After that, the measured *M_t_* can be used to convert *C_ad_* to *C_ar_*.

### 5.5. Model Testing Experiments on Dividing Datasets on Odd and Even Days

We conducted this part of the experiment in two ways. The first method was to train the model using odd-day data and make predictions on even-day data. The quantitative comparison results are shown in Table 7. The second method is to use even-day data for model training and predict odd-day data. The quantitative comparison results are shown in Table 8. When the inputs are eight parameters, MSA-Net has a 20.0% decrease in RMSE compared to MLP (from 7.45 to 5.96). The MSA-Net model reduces RMSE by 7.74% (from 6.46 to 5.96) compared to the Transformer model. In the second method, when the inputs are eight parameters, MSA-Net has an 8.66% decrease in RMSE compared to MLP (from 7.04 to 6.43). The MSA-Net model reduces RMSE by 7.35% (from 6.94 to 6.43) compared to the Transformer model. In addition, MSA-Net has the smallest standard deviation on seven indicators under each partition and different input conditions, indicating good predictive stability. Due to the inconsistent distribution of single- and double-day data, as well as the relatively small proportion of training data to test data, the model learning difficulty is high. Among all comparison models, MSA-Net still achieved the best prediction accuracy, with MAPE of only 0.76% (input four parameters) and 0.87% (input eight parameters).

In summary, within a one-year statistical period, thermal power enterprises can conduct complete data measurements of elemental carbon on odd (even) days and use these measured data for training the MSA-Net model. On even (odd) days, they can measure the *M_t_*, *M_ad_*, *A_ad_*, *V_ad_*, *FC_ad_*, *H_ad_*, *S_t_*_,*ad*_, and *NCV*. With these parameters, the trained MSA-Net can predict the *C_ar_*.

## 6. Discussion

In Section 5.1, we introduced the four commonly used parameters in proximate analysis. When only *M_ad_*, *A_ad_*, *V_ad_*, and *Q_gr,ad_* are used, it contains a total of six parameters including *M_ad_*, *A_ad_*, *V_ad_*, *FC_ad_*, *NCV*, and *H_ad_*. This parameter selection method completely ignores the effect of *S_t,ad_* (from ultimate analysis) on *C_ar_* calculation. If we further increase the parameters *S_t,ad_*, MSA-Net will have a further improvement compared to inputting four parameters as shown in Table 9. However, due to the inherent inclusion of other parameters, effective decoupling is not possible. Therefore, in most cases, MSA-Net with eight inputs can achieve the highest accuracy.

## 7. Conclusions

*C_ar_* is an important parameter for carbon accounting in thermal power enterprises. However, in actual production processes, due to equipment maintenance, repairs, or damage, measurement data may be missing. Using default values will increase the compliance costs of the enterprises. Therefore, it is of great significance to use reliable prediction models to accurately predict *C_ar_* when measurement data are missing, in order to ensure the accuracy of carbon accounting and protect the interests of the enterprise. Based on existing research, this paper first analyzes the parameters related to *C_ar_*. Secondly, these parameters are used as input to propose a carbon content prediction model based on the attention mechanism called MSA-Net. The construction process and details of the Attention module are introduced in detail. Then, the complete measured data are collected from thermal power enterprises, and after data preprocessing, a *C_ar_* prediction dataset is constructed for model training and testing. Subsequently, the effectiveness and reliability of MSA-Net are verified by comparing it with existing methods on the constructed dataset. Finally, two solutions are proposed to reduce the frequency of measurements for thermal power enterprises, thereby reducing their detection costs.

## Figures and Tables

**Table 1:** Parameters of each layer in MSA-Net.

Layer Name		Size
Input		*bs* × *N*
Step-1		
Attention_1	Linear_A1_1	*bs* × *N* × *N*
	Linear_A1_2	*bs* × *N* × *N*
	Linear_A1_3	*bs* × *N* × *N*
Linear_1_1		*bs* × *N* × *M*
Linear_1_2		*bs* × *M* × *N*
Linear_1_3		*bs* × *N* × *N*
Step-2		
Attention_2	Linear_A2_1	*bs* × *N* × *N*
	Linear_A2_2	*bs* × *N* × *N*
	Linear_A2_3	*bs* × *N* × *N*
Linear_2_1		*bs* × *N* × *M*
Linear_2_2		*bs* × *M* × *N*
Linear_2_3		*bs* × *N* × 1
Output		*bs* × 1

**Table 2:** Quantitative evaluation of different models. (RNN, LSTM, MLP, and MSA-Net are the average results).

Input	Method	MAE (×10^3^)	RMSE (×10^3^)	MAPE (%)	R^2^	PCC	CCC	Evar
four parameters	RF [35]	6.49	8.71	1.18	0.8504	0.9233	0.9168	0.8516
	XgbRegressor [36]	5.31	7.40	0.97	0.8921	0.9460	0.9409	0.8936
	SVR [37]	4.98	7.11	0.91	0.9003	0.9552	0.9491	0.9121
	GPR [18]	4.86	6.77	0.89	0.9097	0.9556	0.9509	0.9114
	RNN [33]	5.20	7.07	0.95	0.9015	0.9504	0.9477	0.9030
	LSTM [34]	5.33	7.35	0.97	0.8934	0.9461	0.9432	0.8947
	MLP [19]	5.05	6.91	0.92	0.9059	0.9536	0.9502	0.9081
	MSA-Net	4.73	6.58	0.86	0.9147	0.9567	0.9549	0.9150
eight parameters	RF [35]	6.74	9.49	1.22	0.8225	0.9186	0.8891	0.8231
	XgbRegressor [36]	5.52	7.77	1.00	0.8809	0.9397	0.9339	0.8812
	SVR [37]	5.10	7.04	0.93	0.9024	0.9532	0.9504	0.9078
	GPR [18]	5.02	6.96	0.91	0.9046	0.9540	0.9471	0.9065
	RNN [33]	4.90	6.63	0.89	0.9133	0.9571	0.9544	0.9156
	LSTM [34]	4.92	6.96	0.90	0.9045	0.9532	0.9500	0.9075
	MLP [19]	4.89	6.66	0.89	0.9125	0.9570	0.9540	0.9142
	MSA-Net	4.55	6.20	0.83	0.9242	0.9618	0.9604	0.9176

**Table 3:** Quantitative evaluation of model accuracy and stability.

Input	Method	Statistics	MAE (×10^3^)	RMSE (×10^3^)	MAPE (%)	R^2^	PCC	CCC	Evar
four parameters	RNN [33]	Mean	5.20	7.07	0.95	0.9015	0.9504	0.9477	0.9030
		Std.	0.15	0.09	0.03	0.0024	0.0011	0.0015	0.0021
	LSTM [34]	Mean	5.33	7.35	0.97	0.8934	0.9461	0.9432	0.8947
		Std.	0.16	0.08	0.03	0.0024	0.0014	0.0017	0.0027
	MLP [19]	Mean	5.05	6.91	0.92	0.9059	0.9536	0.9502	0.9081
		Std.	0.28	0.17	0.05	0.0047	0.0018	0.0029	0.0028
	MSA-Net	Mean	4.73	6.58	0.86	0.9147	0.9567	0.9549	0.9150
		Std.	0.09	0.04	0.02	0.0010	0.0007	0.0010	0.0013
eight parameters	RNN [33]	Mean	4.90	6.63	0.89	0.9133	0.9571	0.9544	0.9156
		Std.	0.22	0.06	0.04	0.0014	0.0010	0.0014	0.0021
	LSTM [34]	Mean	4.92	6.96	0.90	0.9045	0.9532	0.9500	0.9075
		Std.	0.15	0.11	0.03	0.0031	0.0011	0.0020	0.0023
	MLP [19]	Mean	4.89	6.66	0.89	0.9125	0.9570	0.9540	0.9142
		Std.	0.15	0.16	0.03	0.0042	0.0023	0.0037	0.0052
	MSA-Net	Mean	4.55	6.20	0.83	0.9242	0.9618	0.9604	0.9176
		Std.	0.18	0.09	0.02	0.0021	0.0011	0.0012	0.0020

**Table 4:** Ablation analysis results of each module.

Method	Huber Loss	Attention Module × 1	Attention Module × 2	Attention Module × 3	Skip-Connections	Input Eight Parameters	MAE (×10^3^)
MLP							5.05
A	√						4.96
B	√	√					4.91
C	√		√				4.87
D	√			√			5.21
MSA-Net	√		√		√		4.73
MSA-Net	√		√		√	√	4.55

**Table 5:** The odd-month data are used for model training, and the even-month data are used for quantitative evaluation of prediction.

Input	Method	Statistics	MAE (×10^3^)	RMSE (×10^3^)	MAPE (%)	R^2^	PCC	CCC	Evar
four parameters	RNN [33]	Mean	5.58	7.40	1.02	0.8779	0.9420	0.9303	0.8814
		Std.	0.17	0.19	0.03	0.0062	0.0028	0.0042	0.0055
	LSTM [34]	Mean	5.68	7.59	1.04	0.8713	0.9392	0.9273	0.8761
		Std.	0.10	0.17	0.02	0.0058	0.0019	0.0047	0.0033
	MLP [19]	Mean	5.22	7.00	0.95	0.8904	0.9470	0.9410	0.8946
		Std.	0.33	0.31	0.06	0.0096	0.0042	.0038	0.0060
	Transformer	Mean	4.94	6.70	0.90	0.8999	0.9508	0.9458	0.9024
		Std.	0.15	0.16	0.03	0.0049	0.0021	0.0038	0.0049
	MSA-Net	Mean	4.81	6.44	0.88	0.9075	0.9537	0.9501	0.9083
		Std.	0.09	0.06	0.02	0.0018	0.0014	0.0013	0.0023
eight parameters	RNN [33]	Mean	5.60	7.49	1.02	0.8746	0.9395	0.9290	0.8774
		Std.	0.23	0.27	0.04	0.0089	0.0054	0.0052	0.0091
	LSTM [34]	Mean	5.95	7.83	1.09	0.8631	0.9337	0.9217	0.8661
		Std.	0.19	0.21	0.03	0.0074	0.0032	0.0064	0.0086
	MLP [19]	Mean	5.30	7.34	0.97	0.8796	0.9419	0.9323	0.8823
		Std.	0.22	0.26	0.04	0.0086	0.0045	0.0063	0.0087
	Transformer	Mean	4.91	6.56	0.90	0.9039	0.9527	0.9481	0.9061
		Std.	0.09	0.15	0.02	0.0043	0.0024	0.0028	0.0041
	MSA-Net	Mean	4.72	6.40	0.86	0.9085	0.9556	0.9505	0.9116
		Std.	0.09	0.07	0.02	0.0019	0.0016	0.0013	0.0030

**Table 6:** The even-month data are used for model training, and the odd-month data are used for quantitative evaluation of prediction.

Input	Method	Statistics	MAE (×10^3^)	RMSE (×10^3^)	MAPE (%)	R^2^	PCC	CCC	Evar
four parameters	RNN [33]	Mean	5.03	6.97	0.90	0.9048	0.9535	0.9515	0.9075
		Std.	0.13	0.12	0.02	0.0034	0.0018	0.0027	0.0029
	LSTM [34]	Mean	5.07	6.97	0.91	0.9048	0.9527	0.9514	0.9057
		Std.	0.17	0.17	0.03	0.0047	0.0022	0.0020	0.0048
	MLP [19]	Mean	4.95	6.98	0.89	0.9043	0.9534	0.9511	0.9063
		Std.	0.34	0.36	0.06	0.0099	0.0043	0.0046	0.0090
	Transformer	Mean	4.59	6.51	0.82	0.9169	0.9597	0.9572	0.9208
		Std.	0.27	0.30	0.05	0.0076	0.0023	0.0037	0.0043
	MSA-Net	Mean	4.31	6.19	0.78	0.9248	0.9622	0.9613	0.9256
		Std.	0.09	0.07	0.02	0.0017	0.0007	0.0008	0.0016
eight parameters	RNN [33]	Mean	4.96	6.72	0.89	0.9115	0.9562	0.9541	0.9121
		Std.	0.19	0.17	0.03	0.0045	0.0020	0.0027	0.0044
	LSTM [34]	Mean	5.50	7.21	0.99	0.8981	0.9495	0.9462	0.9001
		Std.	0.13	0.12	0.02	0.0033	0.0019	0.0032	0.0035
	MLP [19]	Mean	5.30	7.08	0.95	0.9015	0.9547	0.9492	0.9078
		Std.	0.28	0.30	0.05	0.0081	0.0043	0.0060	0.0072
	Transformer	Mean	4.91	6.66	0.88	0.9132	0.9568	0.9557	0.9143
		Std.	0.23	0.16	0.04	0.0042	0.0027	0.0031	0.0040
	MSA-Net	Mean	4.34	6.20	0.78	0.9248	0.9622	0.9613	0.9253
		Std.	0.07	0.08	0.01	0.0019	0.0011	0.0011	0.0020

**Table 7:** The odd-day data are used for model training, and the even-day data are used for quantitative evaluation of prediction.

Input	Method	Statistics	MAE (×10^3^)	RMSE (×10^3^)	MAPE (%)	R^2^	PCC	CCC	Evar
four parameters	RNN [33]	Mean	5.22	7.23	0.94	0.8990	0.9489	0.9470	0.9000
		Std.	0.09	0.09	0.02	0.0025	0.0013	0.0016	0.0026
	LSTM [34]	Mean	5.31	7.33	0.96	0.8963	0.9479	0.9438	0.8971
		Std.	0.09	0.09	0.02	0.0025	0.0012	0.0024	0.0028
	MLP [19]	Mean	5.36	7.46	0.96	0.8921	0.9517	0.9423	0.9021
		Std.	0.39	0.52	0.07	0.0150	0.0047	0.0104	0.0114
	Transformer	Mean	4.54	6.42	0.82	0.9203	0.9599	0.9577	0.9208
		Std.	0.12	0.09	0.02	0.0021	0.0013	0.0012	0.0023
	MSA-Net	Mean	4.37	6.23	0.79	0.9251	0.9627	0.9605	0.9259
		Std.	0.07	0.06	0.01	0.0015	0.0009	0.0013	0.0018
eight parameters	RNN [33]	Mean	5.07	6.94	0.91	0.9070	0.9545	0.9502	0.9096
		Std.	0.21	0.19	0.04	0.0051	0.0020	0.0038	0.0047
	LSTM [34]	Mean	5.63	7.49	1.02	0.8916	0.9460	0.9413	0.8939
		Std.	0.15	0.17	0.03	0.0050	0.0023	0.0026	0.0040
	MLP [19]	Mean	5.48	7.45	0.99	0.8922	0.9507	0.9409	0.8998
		Std.	0.47	0.54	0.08	0.0161	0.0056	0.0107	0.0134
	Transformer	Mean	4.67	6.46	0.84	0.9195	0.9605	0.9568	0.9214
		Std.	0.08	0.11	0.02	0.0028	0.0019	0.0022	0.0045
	MSA-Net	Mean	4.22	5.96	0.76	0.9313	0.9656	0.9642	0.9320
		Std.	0.07	0.06	0.01	0.0014	0.0007	0.0010	0.0014

**Table 8:** The even-day data are used for model training, and the odd-day data are used for quantitative evaluation of prediction.

Input	Method	Statistics	MAE (×10^3^)	RMSE (×10^3^)	MAPE (%)	R^2^	PCC	CCC	Evar
four parameters	RNN [33]	Mean	5.62	7.56	1.02	0.8711	0.9354	0.9297	0.8737
		Std.	0.11	0.09	0.02	0.0029	0.0023	0.0027	0.0043
	LSTM [34]	Mean	5.71	7.60	1.04	0.8696	0.9331	0.9299	0.8703
		Std.	0.11	0.10	0.02	0.0036	0.0018	0.0025	0.0033
	MLP [19]	Mean	5.28	7.10	0.96	0.8862	0.9429	0.9390	0.8877
		Std.	0.18	0.21	0.03	0.0067	0.0032	0.0049	0.0067
	Transformer	Mean	5.13	6.90	0.93	0.8927	0.9467	0.9414	0.8944
		Std.	0.21	0.09	0.04	0.0028	0.0017	0.0019	0.0031
	MSA-Net	Mean	4.98	6.69	0.91	0.8990	0.9486	0.9462	0.8995
		Std.	0.10	0.05	0.02	0.0015	0.0007	0.0015	0.0015
eight parameters	RNN [33]	Mean	5.29	6.98	0.96	0.8901	0.9448	0.9401	0.8914
		Std.	0.12	0.12	0.02	0.0037	0.0019	0.0022	0.0035
	LSTM [34]	Mean	5.73	7.43	1.04	0.8754	0.9378	0.9310	0.8772
		Std.	0.20	0.22	0.04	0.0075	0.0028	0.0056	0.0065
	MLP [19]	Mean	5.25	7.04	0.95	0.8882	0.9447	0.9403	0.8912
		Std.	0.18	0.15	0.03	0.0048	0.0020	0.0040	0.0043
	Transformer	Mean	5.16	6.94	0.94	0.8915	0.9448	0.9416	0.8920
		Std.	0.12	0.08	0.02	0.0026	0.0016	0.0019	0.0027
	MSA-Net	Mean	4.80	6.43	0.87	0.9067	0.9528	0.9508	0.9077
		Std.	0.07	0.06	0.01	0.0017	0.0009	0.0009	0.0017

**Table 9:** The impact of different input parameters on MSA-Net.

Train Sets	Test Sets	Inputs	MAE	RMSE (×10^3^)	MAPE (%)
odd months	even months	*M_ad_*, *A_ad_*, *V_ad_*, *Q_gr_*_,*ad*_	4.81	6.44	0.88
		*M_ad_*, *A_ad_*, *V_ad_*, *Q_gr_*_,*ad*_*, S_t,ad_*	4.75	6.42	0.88
		*M_t_*, *M_ad_*, *A_ad_*, *V_ad_*, *FC_ad_*, *H_ad_*, *S_t_*_,*ad*_, *NCV*	4.72	6.40	0.86
even months	odd months	*M_ad_*, *A_ad_*, *V_ad_*, *Q_gr_*_,*ad*_	4.31	6.19	0.78
		*M_ad_*, *A_ad_*, *V_ad_*, *Q_gr_*_,*ad*_, *S_t,ad_*	4.26	6.10	0.77
		*M_t_*, *M_ad_*, *A_ad_*, *V_ad_*, *FC_ad_*, *H_ad_*, *S_t_*_,*ad*_, *NCV*	4.34	6.20	0.78
odd days	even days	*M_ad_*, *A_ad_*, *V_ad_*, *Q_gr_*_,*ad*_	4.37	6.23	0.79
		*M_ad_*, *A_ad_*, *V_ad_*, *Q_gr_*_,*ad*_*, S_t,ad_*	4.30	6.15	0.78
		*M_t_*, *M_ad_*, *A_ad_*, *V_ad_*, *FC_ad_*, *H_ad_*, *S_t_*_,*ad*_, *NCV*	4.22	5.96	0.76
even days	odd days	*M_ad_*, *A_ad_*, *V_ad_*, *Q_gr_*_,*ad*_	4.98	6.69	0.91
		*M_ad_*, *A_ad_*, *V_ad_*, *Q_gr_*_,*ad*_, *S_t,ad_*	4.85	6.60	0.89
		*M_t_*, *M_ad_*, *A_ad_*, *V_ad_*, *FC_ad_*, *H_ad_*, *S_t_*_,*ad*_, *NCV*	4.80	6.43	0.87

## Data Availability

The original contributions presented in the study are included in the article, further inquiries can be directed to the corresponding author/s.
